# A new species of *Harpactea* (Araneae, Dysderidae) from Aegean region of Turkey

**DOI:** 10.3897/zookeys.59.483

**Published:** 2010-10-01

**Authors:** Kadir Boğaç Kunt, Recep Sulhi Özkütük, Rahşen S. Kaya

**Affiliations:** 1 Turkish Arachnological Society. Eserköy Sitesi 9/A Blok No:7 TR-06530 Ümitköy, Ankara, Turkey; 2 Department of Biology, Faculty of Science, Anadolu University, TR- 26470 Eskişehir, Turkey; 3 Department of Biology, Faculty of Arts and Sciences, Uludağ University, TR-16059, Bursa, Turkey

**Keywords:** Dysderidae, Harpactea, new species, Turkey

## Abstract

A new species of the spider genus Harpactea Bristowe, 1939 is described from the Aegean region of Turkey – Harpactea erseni **sp. n.** (males only). Detailed morphological description and illustrations of the new species are provided. The relationships of the new species are discussed.

## Introduction

The family Dysderidae C. L. Koch, 1837 is represented by 504 species in 24 genera worldwide (Platnick 2010). Harpactea Bristowe, 1939 is a species rich genus with 155 described taxa and is particularly diverse in the Mediterranean region (Platnick 2010). Most of the species appear to be endemics with restricted distributions in the Mediterranean, with only a few found in adjacent areas (Řezáč 2008). So far, 19 species of Harpactea have been recorded from Turkey, 17 of which are endemic to the country (Bayram et al. 2010). However, most of these species are still poorly known and have not been revised since their original descriptions. Nevertheless, the diversity of Harpactea in Turkey is high in comparison to well-studied adjacent countries such as Azerbaijan (14 species), Bulgaria (19 species), Georgia (10 species) and Greece (24 species) (Bosmans and Chatzaki 2005; Otto and Dietzold 2006; Lazarov 2010; Lazarov and Naumova 2010; Van Keer and Bosmans 2010).

During our surveys of the Turkish spider fauna we found one undescribed species of Harpactea in the Aegean region, which is described herein.

## Material and methods

**Figure 1.**
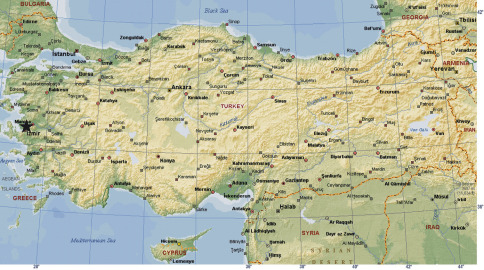
Type locality of Harpactea erseni sp. n (*).

Three males were collected from İzmir province in the Aegean region of Turkey (Fig. 1) using a hand aspirator. The specimens were preserved in 70% ethanol and deposited in the Museum of the Turkish Arachnological Society. Digital images of the pedipalp were taken with a Leica DFC290 digital camera attached to a Leica M205 C stereomicroscope and 5–15 photographs were taken in different focal planes and combined. All measurements are in mm. Terminology for the body measurements and copulatory organ structures were taken from Chatzaki and Arnedo (2006). The following abbreviations were used in the text: AL, abdominal length;CL, carapace length;CWmax, maximum carapace width;CWmin, minimum carapace width;AME, anterior median eyes;PLE, posterior lateral eyes;PME, posterior median eyes;AMEd, diameter of anterior median eyes;PLEd, diameter of posterior lateral eyes;PMEd, diameter of posterior median eyes;ChF, length of cheliceral fang;ChG, length of cheliceral groove;ChL, total length of chelicera (lateral external view);Ta, tarsus;Me, metatarsus,Ti, tibia;Pa, patella;Fe, femur;Tr, trochanter;C, coxa;D, dorsal;Pl, prolateral;Rl, retrolateral;V, ventral;CO, conductor;E, embolus;T, tegulum;MTAS, Museum of the Turkish Arachnological Society, Ankara, Turkey..

## Taxonomy

**Harpactea Bristowe, 1939**

### Harpactea erseni sp. n.

urn:lsid:zoobank.org:act:E8F34578-2D22-4893-A7FC-BD1520B159BA

Figs 2 9 

#### Material examined:

Holotype ♂ (MTAS) **İzmir Province**, Yamanlar Mountain, Karagöl [38°33'26.00N; 27°13'11.00E], 28. XI. 2008, under stones, leg. K.B.Kunt. Paratypes: 2 ♂ (MTAS) same data as holotype.

#### Derivatio nominis:

The new species is dedicated to “Ersen Aydın Yağmur” who made a great contribution to our knowledge of Turkish scorpions and who is a good friend of the authors.

#### Diagnosis:

Harpactea erseni sp. n. differs from other Turkish Harpactea species (see Nosek 1905; Alicata 1974; Brignoli 1978a-b; Brignoli 1979; Bayram et al. 2009) in the structure of the pedipalp of the male. However, the palpal structures of Harpactea erseni sp. n. are close to Harpactea strandjica Dimitrov, 1997 and Harpactea terveli Lazarov, 2009 described from Bulgaria (see Lazarov 2009). The new species can be distinguished from Harpactea terveli by the different shape of the embolus and conductor; and from Harpactea strandjica by having a thinner, curved embolus without a bifurcated tip.

#### Comments:

Harpactea is one of the most endemic and speciose dysderid genera in Turkey, with 17 endemics (Bayram et al. 2010). Most of the endemic species have restricted distributions and occur at high elevations, such as the mountain ranges of the Mediterranean, and the north and central Anatolian regions. This distribution pattern presumably results from the combination of topography, proximate biogeographical subregions, the high number of different biotopes and the climate of Anatolia, all of which play a special role in speciation. In short, the Anatolian Harpactea fauna is characterized by a high level of local endemism, and by limited co-occurrence of species in the adjacent zoogeographical regions. However, one question can be raised regarding the newly described species: is the male a specimen of a previously described species known only from the female (presumably from Turkey or neighboring countries)? According to our morphometric data, our samples are larger than all previously described Harpactea species from Turkey (see Brignoli 1978a–b), supporting our conclusion that it is in fact a new species, rather than the male of a previously described female.

#### Measurements (holotype):

**AL** 4.05; **CL** 3.45; **CWmax** 2.25; **CWmin** 1.35; **AMEd** 0.15; **PLEd** 0.13; **PMEd** 0.10; **ChF** 0.76; **ChG** 0.34; **ChL** 1.35 mm. Leg measurements are given in Table 1.

**Table 1.** Leg measurements of Harpactea erseni sp. n.

Legs	I	II	III	IV
**C**	1.65	1.28	0.88	1.20
**Tr**	0.52	0.34	0.34	0.37
**Fe**	3.15	2.63	2.45	3.00
**Pa**	1.20	1.76	1.23	1.50
**Ti**	2.55	2.46	2.02	3.38
**Me**	2.63	2.25	2.15	2.63
**Ta**	0.80	0.83	0.83	0.84
**Total**	12.50	11.55	9.90	12.92

#### Description:

**Figures 2–5.**
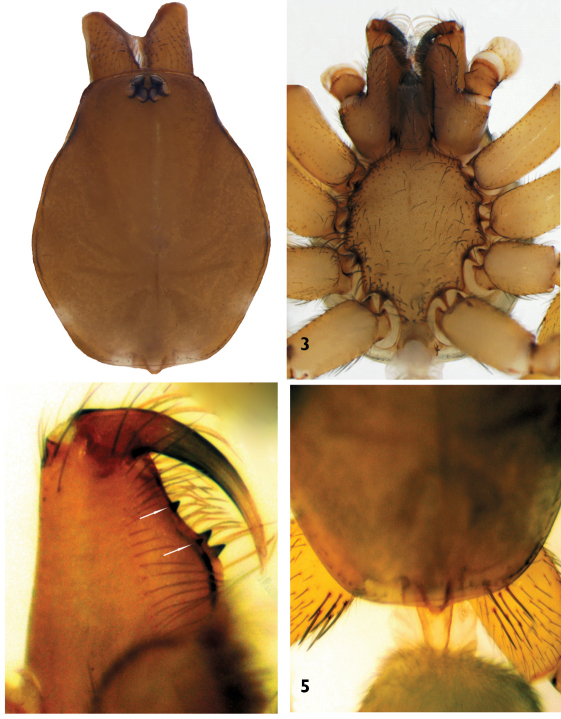
Harpactea erseni sp. n. **2** Carapace **3** Sternum **4** Right chelicer, ventral view **5	** Coxae IV, dorsal view.

Carapace light brown, with smooth surface and distinct fovea. AME, PLE and PME closely grouped; AME separated (Fig. 2). Sternum, labium, gnathocoxae and chelicerae yellowish-brown. Sternum with long, thin hairs near the margin (Fig. 3). Cheliceral groove with four teeth: retromargin with two teeth, including a tiny one at the base of the groove; promargin with two strong teeth of equal size close to each other. Top of the labium and gnathocoxae with short, strong hairs, sparsely distributed (Fig. 4). Abdomen greyish-light brown, with short, thin blackish hairs over the entire surface. Legs yellowish-light brown with sparse blackish setae. Leg IV > Leg I > Leg II > Leg III. Tarsi with three claws. Tarsi III and IV with fine scopulae. Legs III and IV with fine metatarsal scopulae covering slightly less than the distal half of the segment (ventral surface only). Dorsal part of coxae III and IV with 2–6 spines (Fig. 5). Details of leg spination are given in Table 2.

**Figures 6–9.**
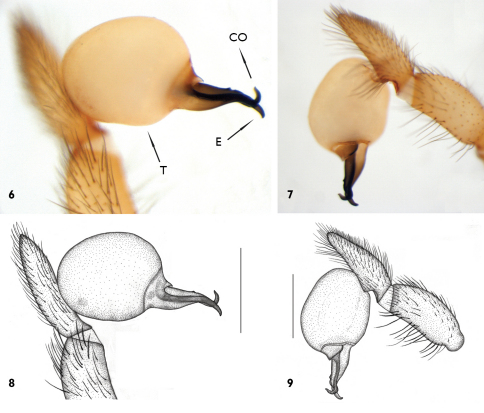
Harpactea erseni sp. n., general appearance of right bulb **6–8** Prolateral view **7–9** Retrolateral view **CO** Conductor **E** Embolus **T** Tegulum (Scale lines: 0.5 mm)

Palpal organ with globular bulb and curved, black embolus tapering towards the tip. Conductor same colour as embolus and hook-shaped at the tip and with a tuberculum on the mid-part (Figs 6–9). Female unknown.

**Table 2.** Leg spination of Harpactea erseni sp. n.

Legs	I	II	III	IV
**C**	0	0	2-3 D	5-6 D
**Tr**	0	0	0	0
**Fe**	2 Pl	1, 1 Pl	2, 2, 2 D	1, 2, 2 D, 2-4 Rl
**Pa**	0	0	1 D	1 Rl
**Ti**	0	0	6 Pl, 1 Rl, 2, 1, 2 V	1, 1, 1 Pl, 1, 1 Rl, 5-6 V
**Me**	0	0	6 Pl, 1, 1 Pl, 5-6 V	3 Pl, 4 Rl, 3-5 V
**Ta**	0	0	0	0

## Discussion

According to the classification of Deeleman-Reinhold (1993), Harpactea erseni sp. n. belongs in the *rubicunda* (D) species group which is characterized by having a globular palpal body, a massive embolus and conductor and patellae-coxae with spines. Up until now 20 (including the new species) Harpactea species have been reported from Turkey. Although the diversity of this genus in Turkey is comparatively high, it can be expected that the actual diversity will be even higher because many regions with favourable habitats for Harpactea remain to be studied in Turkey. Therefore, we expect that more species will be found in the future.

## Supplementary Material

XML Treatment for Harpactea erseni
